# Single-Cell RNA-Sequencing Reveals Heterogeneity and Transcriptional Dynamics in Porcine Circulating CD8^+^ T Cells

**DOI:** 10.3390/cells13080692

**Published:** 2024-04-16

**Authors:** Pingping Han, Yaping Guo, Wei Zhang, Daoyuan Wang, Yalan Wu, Xinyun Li, Mengjin Zhu

**Affiliations:** 1 Key Laboratory of Agricultural Animal Genetics, Breeding, and Reproduction of Ministry of Education, Huazhong Agricultural University, Wuhan 430070, China; hping@webmail.hzau.edu.cn (P.H.); ypguo@webmail.hzau.edu.cn (Y.G.); zzymyyds@outlook.com (W.Z.); dywang.123@webmail.hzau.edu.cn (D.W.); wuyalan@yingzigene.com (Y.W.); xyli@mail.hzau.edu.cn (X.L.); 2 The Cooperative Innovation Center for Sustainable Pig Production, Huazhong Agricultural University, Wuhan 430070, China

**Keywords:** pig, immunity, CD8 T cell, heterogeneity, pseudotime trajectory

## Abstract

Pigs are the most important source of meat and valuable biomedical models. However, the porcine immune system, especially the heterogeneity of CD8 T cell subtypes, has not been fully characterized. Here, using single-cell RNA sequencing, we identified 14 major cell types from peripheral blood circulating cells of pigs and observed remarkable heterogeneity among CD8 T cell types. Upon re-clustering of CD8^+^ T cells, we defined four CD8 T cell subtypes and revealed their potential differentiation trajectories and transcriptomic differences among them. Additionally, we identified transcription factors with potential regulatory roles in maintaining CD8 T cell differentiation. The cell-cell communication analysis inferred an extensive interaction between CD8 T cells and other immune cells. Finally, cross-species analysis further identified species-specific and conserved cell types across different species. Overall, our study provides the first insight into the extensive functional heterogeneity and state transitions among porcine CD8 T cell subtypes in pig peripheral blood, complements the knowledge of porcine immunity, and enhances its potential as a biomedical model.

## 1. Introduction

CD8 T lymphocytes, also known as cytotoxic T lymphocytes (CTLs), are responsible for eliminating intracellular pathogens and combating tumor cells, and they play an essential role in the adaptive immune system. CD8 T cells recognize peptide antigens on the surface of antigen-presenting cells through their T cell receptor (TCR) in the context of major histocompatibility complex (MHC) class I molecules [1]. Upon receiving antigen signals and under the actions of co-stimulatory molecules and cytokines, naive CD8 T cells undergo proliferation and differentiation to generate effector CD8 T cells with cytotoxic activity [2]. CTLs primarily employ two pathways, including the release of cytotoxic effector molecules (such as perforin, granzymes, and Fas ligand), and the production of effector cytokines (such as IL-2, TNF, and IFN-γ) to eliminate target cells [3]. Several studies have indicated that during viral infections, such as classical swine fever virus (CSFV), foot-and-mouth disease virus (FMDV), and porcine reproductive and respiratory syndrome virus (PRRSV), the presence of a high proportion of CTLs contributes to enhancing immunity and promoting infection recovery [4,5,6]. Therefore, a comprehensive understanding of the differentiation and functionality of CD8 T cells is essential for optimizing immune therapeutic strategies and future vaccine development.

The differentiation stages and phenotypic functions of CD8 T cells are well characterized in the human immune system. According to the expression of monoclonal antibodies CD45RA, CD28, and CCR7, human CD8 T cells are further divided into four subtypes, namely, naive (CD45RA^+^CD28^+^CCR7^+^), Tem (CD45RA^−^CD28^+^CCR7^−^), Tcm (CD45RA^−^CD28^+^CCR7^+^), and Temra (CD45RA^+^CD28^−^CCR7^−^) [7,8,9]. These four subtypes exhibit distinct phenotypic and functional characteristics, playing crucial roles in the different stages of the immune response. For instance, Tcm cells residing in secondary lymphoid organs typically exhibit higher expressions of proliferation-related genes compared to Tem cells. Conversely, Tem cells with effector functions exhibit higher expression of effector molecules, such as perforin and granzymes [10,11].

As pigs are increasingly popular as a large animal model for preclinical research and an important food source, it is crucial to gain a deeper understanding of pig immune characteristics, particularly those associated with human immune cells. Despite some similarities between the human and porcine immune systems, the characterization of porcine CD8 T cells is still limited. Early studies of porcine immune cells rely primarily on antibody-based assays, and porcine CTLs are typically characterized using expression of the lineage markers CD3, CD8α, and CD8β [12]. Afterward, based on the expression levels of CD27, SLA-DR, and perforin, porcine CTLs are classified into early effector cells (CD3^+^CD8αβ^+^perforin^+^CD27^dim^) and late effector or memory cells (CD3^+^CD8αβ^+^perforin^+^CD27^−^) [13]. Recently, in combination with surface antigen-based cell sorting and bulk RNA sequencing, porcine CTLs have been classified into naive cells (CD8β^+^CD27^+^CD11a^low^), intermediate differentiated cells (CD8β^+^CD27^dim^CD11a^+^), and terminally differentiated cells (CD8β^+^CD27^−^CD11a^high^), according to the expressions of CD8β, CD27, and CD11a [1]. Although these studies contribute to our understanding of CD8 T cell heterogeneity, certain limitations exist. Firstly, pre-selection bias potentially arises in the case of limited antibody availability or the use of excessive markers to define cell populations, since different CD8^+^ T cell phenotypes are typically defined by combining multiple specific cell surface markers. Secondly, flow cytometric analysis tends to detect only a limited number of cells, thus potentially impeding the accurate classification of different T cell phenotypes. Finally, bulk RNA sequencing, which is conducted at the population level, provides only average gene expression profiles, and it cannot capture subtle intercellular variations or track dynamic changes in individual cells due to a lack of high resolution.

Currently, the rapid development of single-cell RNA sequencing (scRNA-seq) technology makes it possible to comprehensively characterize cellular heterogeneity and explore the differentiation trajectory of the porcine immune cells. In this study, we characterized the composition of immune cells in pig peripheral blood, analyzed gene expression signatures, pathway patterns, developmental trajectory, and gene regulatory networks of CD8 T cell subtypes, inferred intercellular communication between CD8 T cell and other cell types using scRNA-seq technology. This study will increase the understanding of porcine CD8 T cell heterogeneity and contribute to the further exploration of the porcine immune system.

## 2. Materials and Methods

### 2.1. Sample Collection and Processing

Peripheral blood mononuclear cells (PBMCs) were collected from three ~7-month-old healthy Large White sows at the experimental pig farm at Huazhong Agricultural University. Peripheral blood samples (2 mL) were collected into an EDTA anticoagulant tube and gently inverted 2–3 times to ensure thorough mixing. Subsequently, the mixed blood samples were transferred to a 15 mL centrifuge tube and treated with 6 mL of red blood cell lysis solution for 15 min, followed by centrifugation at 1600 rpm for 10 min at 4 °C to collect cell pellets. The cell pellets were then resuspended in cold phosphate-buffered saline (PBS), passed through a 100 μm cell strainer, and incubated with 3 μL CD3ε antibody (clone BB23-8E6-8C8, BD Pharmingen, San Diego, CA, USA) for 30 min at 4 °C in the dark, followed by FACS to obtain living CD3^+^ PBMCs. The cell viability assay used trypan blue staining. After cell counting, the samples from the three healthy pigs were mixed in equal volumes to obtain 9 × 10^5^ cells. The mixed cells were used for constructing a 10× genomics sequencing library and subsequent sequencing by Genergy Biotechnology (Shanghai, China).

### 2.2. Processing of scRNA-Seq Data

Considering that large datasets contribute to enhancing the cell resolution in scRNA-seq analysis, we downloaded seven publicly available datasets additionally and integrated them with our experimentally generated datasets to dissect peripheral blood heterogeneity [14]. The initial processing of public scRNA-seq data, including reads 2 (R2) correction, 3′polyA tail trimming, read alignment, and gene quantification and ambient RNAs removal, was conducted by the methods described by Herrera-Uribe et al. [14]. Subsequently, low-quality cells (with <500 detected genes and <1000 unique molecular identifiers (UMIs) per cell and mitochondrial gene content per cell >10%) were excluded from each sample. To further refine the dataset, the Scrublet python package (v0.2.3) was employed to identify and remove doublet cells. A preset doublet formation rate of 0.07 was used for six samples from public databases, while a doublet cell formation rate of 0.06 was applied for samples generated in this study and other samples from public databases. The cells with a doublet probability score >0.25 were removed. The filtered data were stored in CellRanger format.

### 2.3. Identification of Cell Clusters

After quality control and doublet cell removal, data from eight PBMC samples were integrated using the Seurat package (v4.3.0.1). Subsequently, we performed data normalization employing the NormalizeData function and identified 3000 highly variable genes (HVGs) using the FindVariableFeatures function with the vst method. Further, we integrated and batch-corrected the datasets following the standard integration workflow using the Seurat package with default parameters (https://satijalab.org/seurat/archive/v3.0/integration.html, accessed on 8 January 2023). To address variability induced by UMIs and mitochondrial genes, we performed regression analysis using the vars.to.regress argument in the ScaleData function. Expression levels of all genes were scaled for subsequent principal component analysis. Further, all the cells were subjected to cluster analysis to obtain cell clusters, with dimension set as 20 and resolution set as 1.8. The cell clusters were visualized using Unified Manifold Approximation and Projection (UMAP). The cell clusters were classified and annotated based on the expressions of cell type-specific canonical markers. T cell re-clustering was performed by the above-mentioned methods, except that the dimension was set to 8 and the resolution was set to 0.7. Notably, several gene names/Ensembl IDs used for data analysis in this study were replaced with updated ones due to their unavailability in the annotation file, as previously described [14].

### 2.4. Differential Gene Expression Analysis

In this study, we employed two different methods for the analysis of differentially expressed genes (DEGs). One method was the Wilcoxon rank sum test using the FindAllMarkers function in the Seurat package, and the other method was pseudobulk conversion using the method described by Ammons et al. [15]. Specifically, the genes with <3 raw counts across all sample cells were removed, and only those genes that were expressed in more than 5 cells within a sample were included in the pseudobulk conversion. Subsequently, we applied the DESeq2 (v1.34.0) pipeline to identify DEGs between the target cluster and other groups [16]. In the pseudobulk conversion method, DEGs were identified from adjusted thresholds of *p* < 0.05 and a |log_2_ FC (fold change)| > 0.58. In the Wilcoxon rank sum test method, DEGs were identified using the FindAllMarkers function with the parameters only.pos = T, logfc.threshold = 0.25, and min.pct = 0.25.

### 2.5. GO Enrichment Analyses

**Figure 1:**
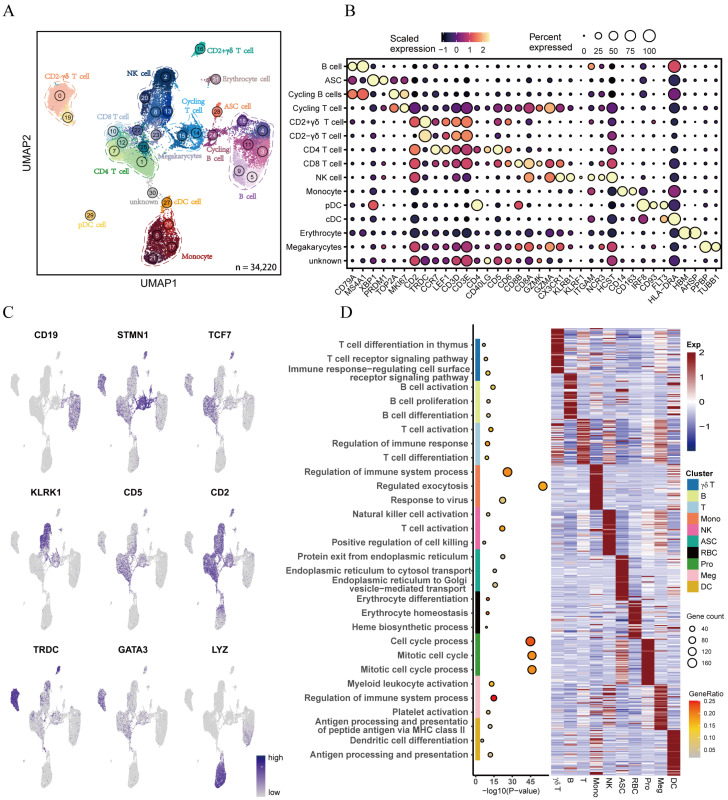
Single-cell transcriptomic analysis of porcine peripheral blood mononuclear cells. (**A**) Unified manifold approximation and projection (UMAP) visualization of 34,220 cells from 8 PBMC samples. Each color represents one cell cluster. (**B**) Dot plot of the expression of cell type-specific marker genes. Dot brightness and size represent the scaled expression of each marker gene and the proportion of cells expressing each marker gene, respectively. (**C**) UMAP plots of marker genes used for defining cell types. (**D**) GO enrichment analysis of DEGs. The left panel presents GO terms enriched in 10 major cell types. The right panel is the heatmap of the top 50 DEGs (Wilcoxon test) in each of the 10 major cell types. The gene expression value is a row-scaled Z score.

DEGs in the 10 major cell types were identified using the Wilcoxon rank sum test (only.pos = T, min.pct = 0.25, logfc.threshold = 0.25) (Figure 1D). The DEGs in the 10 major cell types were subsequently subjected to gene ontology (GO) analysis (http://metascape.org, accessed on 30 January 2023) [17]. Go terms with *p* < 0.01, a minimum count of 3, and an enrichment factor >1.5 were considered significantly enriched. DEGs in CD4 T clusters and γδ T clusters were identified using the pseudobulk conversion method (adjusted *p* < 0.05 and |log_2_ FC| > 0.58). GO analysis of these DEGs was performed using the enrichGO function within the clusterProfiler package (v4.2.2) [18].

### 2.6. Gene Set Variation Analysis of CD8 T Cell Subtypes

To compare the functional profiles of different CD8 T cell subtypes, we employed the Wilcoxon rank-sum test for DEG identification (only.pos = T, logfc.threshold = 0.25) and the compareCluster function in the clusterProfiler package (v4.2.2) for GO analysis of identified DEGs. Gene set variation analysis (GSVA) was performed using the GSVA package (v1.42.0) [19]. The annotated gene sets, including H (hallmark gene sets) and C2 (CP: KEGG gene sets), were downloaded from the Molecular Signatures Database (MSigDB, v7.0).

### 2.7. Pseudotime Trajectory Analysis

The Monocle2 package (v2.22.0) was used to infer single-cell developmental trajectories of CD8 T cells, following the standard analysis pipeline (http://cole-trapnell-lab.github.io/monocle-release/docs/#constructing-single-cell-trajectories, accessed on 1 March 2023) [20]. We selected 3000 HVGs identified by the function FindVariableFeatures in Seurat to sort cells in pesudotime order. We then performed dimensionality reduction in cells by the DDRTree method and ordered the cells with the orderCells function. Finally, “plot_cell_trajectory” and “plot_pseudotime_heatmap” were used to visualize pseudo-time gene branching trajectories and plot the heatmaps of marker genes in CD8 T cell subtypes. Additionally, we applied the Slingshot package (v2.2.1) to compute pseudotime trajectories of CD8 T cells and mapped these trajectories onto the UMAP for visualization [21].

### 2.8. Cell-Cell Communication Analysis with CellPhoneDB

Cell-cell communication between different cell types was conducted using the CellPhoneDB software (v2.1.7), as previously described [22]. Gene expression matrices and metadata containing major cell annotations were input into CellPhoneDB with default parameters for subsequent analysis. The strength of cell-cell interactions was visualized using a heatmap, and the cell-cell interactions mediated by putative receptor-ligand pairs were presented using a dot plot.

### 2.9. Transcription Factor Analysis

We extracted the expression profiles of all CD8 T cells and input them into SCENIC (single-cell regulatory network inference and clustering, v1.3.1) to construct a gene regulatory network [23]. The transcription factor (TF) data were analyzed based on the “hg19” dataset, with the search region limited to 10 kb around the transcription start site (TSS) or 500 bp upstream of the TSS. The SCENIC analysis (corresponding to RcisTarget v1.12.0 and AUCell v1.14.0) was performed following the pipeline described by Gao et al. (https://github.com/YahGao/Rumen-scRNA-seq/blob/master/4_scenic.R, accessed on 10 April 2023) [24]. To further validate the reliability of these identified TFs in CD8 T cells, we combined the reported scATAC-seq data of PBMC and integrated the enriched TFs identified by scATAC-seq data with those identified by our scRNA-seq data [25].

### 2.10. Porcine-Human and Porcine-Canine Homology Analysis

We performed a cross-species comparison by integrating previously published and fully annotated scRNA-seq datasets. These datasets included scRNA-seq data of six healthy adult human PBMCs (https://zenodo.org/record/4021967/, blish_covid.seu.rds, accessed on 30 May 2023) and seven healthy dog PBMCs (https://github.com/dyammons/Canine_Leukocyte_scRNA/tree/main/input, GSE225599_final_dataSet_H.rds, accessed on 30 May 2023), which were integrated with the scRNA-seq data of eight healthy pig PBMCs in this study [14,15,26]. First, each sample data was independently normalized using the SCTransform function in the Seurat package (v4.3.0.1), and the regression analysis of mitochondrial gene content was performed. Subsequently, the SelectIntegrationFeatures function was applied to identify the top 2000 HVGs that consistently changed across the datasets. Integration anchors were then identified within the dataset using the FindIntegrationAnchors function. Next, the IntegrateData function was employed to integrate pig and human datasets or pig and dog datasets. After integration, cell types of pig, human, and dog were prefixed with “pig_”, “hu_”, or “dog_”, respectively. Hierarchical clustering analysis was carried out using the hclust function.

## 3. Results

### 3.1. Transcriptional Landscape Reveals Heterogeneity of Porcine Peripheral Blood Monocytes

To investigate the intrinsic structure and potential functional subtypes of peripheral immune cells, we performed a 10× Genomics single-cell RNA sequencing on a mixed population of CD3^+^ PBMCs isolated from three healthy Large White pigs. Considering that cell resolution of scRNA-seq analysis can be improved based on large datasets, we also downloaded seven additional PBMC scRNA-seq datasets generated by Herrera-Uribe et al. using the same technique (10× Genomics) [14]. After rigorous quality control and the removal of batch effects, a total of 14,595 transcriptomes were obtained from 34,220 cells in eight PBMC samples for subsequent analyses. Using the graph-based uniform manifold approximation and projection (UMAP) clustering algorithm in the Seurat package, we identified 32 cell clusters (Figure 1A and Figure S1A–C). These cell clusters were further classified into 14 main cell types based on classical marker gene expression (Figure 1A–C and Figure S2F). These cell types included B cells (cluster 3, 4, 5, 9, 11, 18), CD2^−^γδ T cells (cluster 0, 19), CD2^+^γδ T cells (cluster 16), CD4 T cells (cluster 1, 7, 12, 25), CD8 T cells (cluster 8, 10, 13, 22), NK cells (cluster 2, 20), antibody-secreting cells (ASCs; cluster 28), erythrocytes (cluster 31), plasmacytoid dendritic cells (pDCs; cluster 29), conventional dendritic cells (cDCs; cluster 27), and monocytes (cluster 6, 17, 21, 26). These cell types were consistent with those identified based on published scRNA-seq data in a previous study [14]. We additionally identified megakaryocytes (cluster 23, *PPBP*, *TUBB1*, *ITGA2B*, *ITGB3*), proliferative T cells (cluster 14, 15; *MKI67*, *TOP2A*, *STMN1*), and proliferative B cells (cluster 24; *MKI67*, *TOP2A*, *CD79A*, *MS4A1*). Cluster 30 included multiple cell types highly expressing various marker genes, indicating that cluster 30 was a mixed population, and thus cluster 30 was excluded from subsequent analyses. The proportions of each cell type are shown in Figure S2F. To validate the above-mentioned 14 cell types, we utilized the reference mapping of the human database in the SingleR package (v1.8.1) (Figure S2A–E) [27]. Unfortunately, some of these 14 cell types of pig did not well match those of the human, especially T cells. This mismatch might be attributed to the inadequate annotation of pig immune-related genomes and interspecies variations.

To further explore the potential functions of these major cell types, we performed functional enrichment analysis of the DEGs in each major cell type using metasacpe (Figure 1D). The enrichment analysis results effectively confirmed the accuracy of the categorization of these cell types. For instance, GO terms assigned to B cells included “B cell activation”, “B cell proliferation”, and “B cell differentiation”. Meanwhile, proliferation cells exhibited enrichment in such GO terms as “cell cycle process”, “mitotic cell cycle”, and “mitotic cell cycle process”. GO terms immune-regulatory and T cell activation were assigned to T cells and γδ T cells. NK cells showed the enrichment of GO terms related to “cell killing”. Dendritic cells (pDCs and cDCs) displayed the enrichment of GO terms, such as “Antigen processing and presentation of peptide antigen via MHC class II”, “Dendritic cell differentiation”, and “Antigen processing and presentation”, which was consistent with previous reports by Herrera-Uribe et al. [14]. Notably, some genes were expressed in both T cell cluster and NK cell cluster, suggesting similar expression patterns in these two clusters. Collectively, we identified 14 different cell types and characterized their gene-expression signatures, and our results preliminary suggested that peripheral blood cells were highly heterogeneous.

### 3.2. Clustering Analysis Reveals Heterogeneity of T Lymphocytes in Peripheral Blood

**Figure 2:**
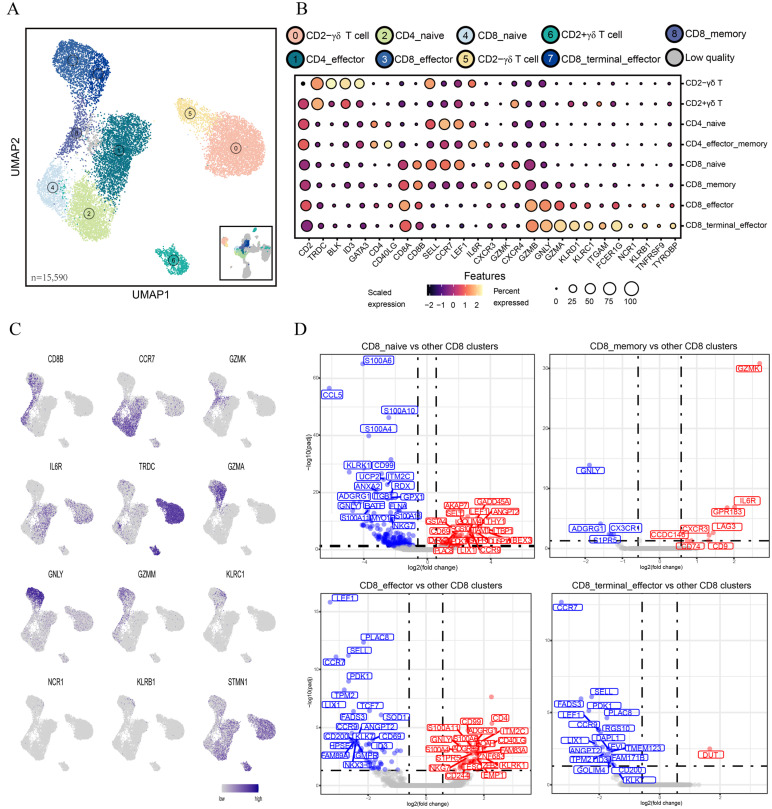
Transcriptomic heterogeneity of CD8 T lymphocytes. (**A**) UMAP visualization of T cell subtypes. Different colors represent different cell clusters. (**B**) Dot plot of the expression of cell type-specific marker genes. Dot brightness and size represent the scaled expression of each marker gene and the proportion of cells expressing each marker gene, respectively. (**C**) UMAP plots of marker genes used for defining cell types. (**D**) Volcano plot of the DEGs (pseudobulk conversion) between the target CD8 cluster and all other CD8 clusters. Adjusted *p* values < 0.05 and |log_2_ FC| > 0.58 were used as DEG screening criteria. Red and blue dots denote up-regulated and down-regulated DEGs, respectively.

Our data showed that T lymphocytes, the most important immune cells in anti-viral response, dominated in peripheral blood. Therefore, we further explored the transcriptional heterogeneity of T lymphocytes. Based on *TRDC* gene expression, we divided nine PBMC-derived T cell clusters obtained from unsupervised clustering into two cell lineages, namely, αβ T cell lineage (9749 T cells) and γδ cell lineage (5841 T cells) (Figure 2A and Figure S3A,B). The αβ T cell lineage was further divided into CD4^+^ (4145) and CD8^+^ (3984) T cells based on the expression of *CD4*, *CD8A*, and *CD8B*, and subsequently, CD4^+^ and CD8^+^ T cells were subdivided into two and four cell subtypes, respectively (Figure 2B,C). The γδ cell lineage was divided into CD2^+^γδ T cells (728) and CD2^−^γδ T cells (5113) based on *CD2* expression levels. The identity of one cell cluster could not be determined, and this unknown cell cluster was excluded from downstream analysis. Among CD8 T cells, cluster 4 exhibited specific high expression of naive markers, such as *LEF1*, *CCR7*, and *SELL*, clearly confirming its identity as CD8_naive. Cluster 8 displayed the specific expression of *GZMK*, *CXCR4*, and *CXCR3*, commonly associated with memory functions of T cells, and thus, cluster 8 was designated as CD8_memory. Notably, cluster 3 exhibited high expression levels of effector marker genes, including *GZMM*, *GZMB*, *CCL5*, and *GNLY*, and thus, cluster 3 was defined as CD8_effector. The remaining CD8 T cells were assigned to cluster 7, and cluster 7 shared several genes with cluster 3, exhibiting high expression of several cytotoxic genes and exhaustion molecules, including *ITGAM*, *KLRD1*, *TNFRSF9*, and *TYROBP*, implying that the identity of cluster 7 was CD8_terminal_effector cells. In addition to our manual classification, we conducted differential gene expression analysis using pseudobulk conversion by previously reported methods to further define each key cell type [15]. In line with our manual classification results, we found that the CD8_naive cluster displayed higher expression of naive-associated genes (*LEF1*, *CCR7* and *SELL*) and lower expression of cytotoxic genes (*GZMB*, *NCR3*, and *KLRK1*), relative to all other CD8 T cells (Figure 2D, Table S1). CD8_effector and CD8_terminal_effector T cells displayed higher expression of effect-related and cytotoxicity-related genes, such as *LYZ* (lysozyme), *S100A11* (alarmin), *NCR1* than other CD8 T cells (Figure 2D, Table S1).

Similarly, we identified two CD4 T cell subtypes (cluster 2 and cluster 1) (Figure 2B,C). Cluster 2 was characterized by the high expression of naive marker genes, such as *CCR7* and *LEF1*, indicating that cluster 2 was CD4_naive T cells, whereas cluster 1 was characterized by the high expression of activation-associated markers (*ITGB1*, *CD40LG*, *IL6R*), suggesting that cluster 1 was activated CD4^+^αβ T cells, and thus, cluster 1 was designated as CD4_effector_memory (CD4_TEM), which was in agreement with previous findings [14]. Further differential gene expression analysis based on pseudobulk conversion revealed upregulated expression of several naive genes in CD4_naive T cells and activity-related genes in CD4_TEM (Figure S3C, Table S2). GO enrichment analysis of DEGs obtained by the pseudobulk conversion method confirmed functional differences between CD4_naive and CD4_TEM. CD4_naive T cells were mainly involved in several biological pathways related to growth, development, cell regulation, and tissue formation in organisms, such as the regulation of the cell cycle pathway and the transmembrane receptor protein tyrosine kinase signaling pathway. CD4_TEM cells were associated with the regulation of lymphocyte activation and cell adhesion (Figure S3D, Table S3).

Pigs are considered a high γδ species. In this study, we identified two major subtypes of γδ T cells, namely, CD2^+^γδ and CD2^−^γδ cells (Figure 2B,C). Our results were consistent with previous reports that CD2^−^γδ T cells dominate in porcine blood [28,29]. Further, we performed pseudobulk conversion on the γδ cell population and found that CD2^+^γδ T cells exhibited high expression levels of genes related to TCR signaling (*PRKCH*, *LCK*, and *IKZF2*), whereas CD2^−^γδ T cells displayed the expression of the *JAML* gene (Figure S3E, Table S2). This finding is in close agreement with the porcine thymus single-cell data reported by Gu et al., suggesting that a portion of the γδ T cells in the blood might have originated from the thymus [30]. Our GO enrichment analysis revealed that CD2^−^γδ T cells were mainly involved in the positive regulation of response to stimulus and the regulation of immune system process and that CD2^+^γδ T cells demonstrated activity in the process of cell positioning (such as cell migration and cell motility) (Figure S3F, Table S3).

### 3.3. Differences in Functions and Immunometabolic Patterns among CD8 T Cell Subtypes

**Figure 3:**
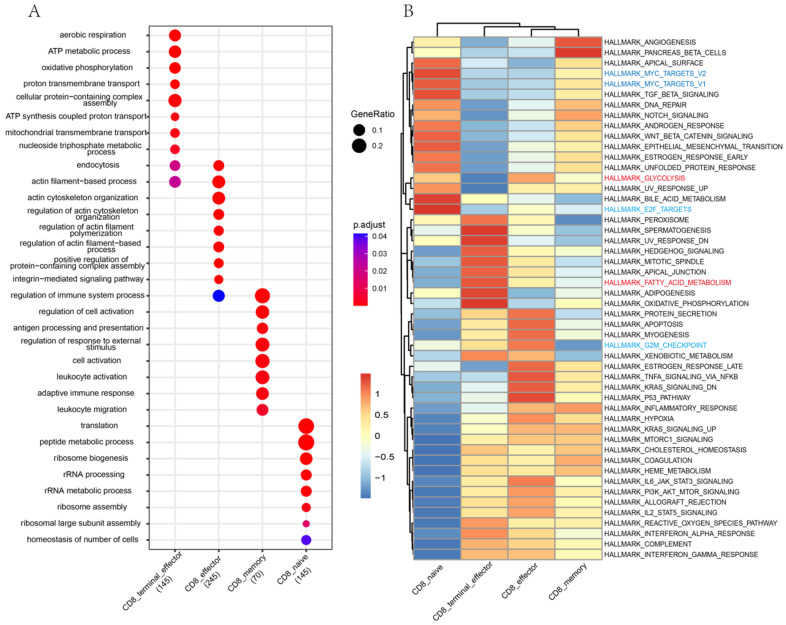
Pathway heterogeneity among CD8 T cell subtypes. (**A**) GO analysis of biology processes in each CD8 T cell subtype. Node size represents the number of genes, and color intensity corresponds to the adjusted *p*-value. (**B**) GSVA heatmaps of hallmark gene sets in each of 4 CD8 T cell subtypes. The important metabolic gene sets are highlighted in red, while the cell cycle-related gene sets are highlighted in blue.

To investigate the function heterogeneity among different CD8 T cell subtypes, we conducted GO analysis, pathway activity analysis, and Kyoto Encyclopedia of Genes and Genomes (KEGGs) analysis. GO analysis revealed that CD8_naive T cells were mainly related to the ribosome synthesis-related pathway, CD8_memory and CD8_effector cells were primarily associated with immune response, and CD8_terminal_effector T cells were involved in ATP metabolism and aerobic respiration processes (Figure 3A). Additionally, GSVA results indicated substantial heterogeneity among CD8 T cell subtypes (Figure 3B). In detail, CD8_naive cells displayed relatively low activity in fatty acid metabolism but relatively high activity in cell proliferation and differentiation, such as MYC targets v1/v2, TGF-β signaling, E2F targets, and WNT/β catenin signaling. CD8_memory T cells exhibited a similar low activity in fatty acid metabolism. PI3K/Akt/mTOR signaling pathway, glycolysis, and several immune activation-related pathways (interferon-γ response, IL2-STAT5 signaling, TNFA signaling via NFκB, and IL6-JAK-STAT3 signaling) were highly activated in CD8_effector. Notably, both inflammatory response and mTORC1 signaling were relatively active in CD8_memory and CD8_effector. Furthermore, three pathways, including fatty acid metabolism, oxidative phosphorylation, and adipogenesis, showed the highest activity in the CD8_terminal_effector T cells. Consistent with the pathway activity analysis results, the KEGG results revealed that CD8_naive T cells were mainly related to the cell cycle pathway, whereas CD8_memory T cells were primarily associated with valine, leucine, and isoleucine biosynthesis, as well as glycolipid metabolism (Table S4). The immune-inflammation pathways exhibited high activity in CD8_effector T cells, while fatty acid metabolism and oxidative phosphorylation pathways displayed high activities in CD8_terminal_effector T cells. Taken together, our results revealed complex function heterogeneity among CD8 T cell subtypes.

### 3.4. Pseudotime Trajectory Analysis Reveals Dynamic Heterogeneity among CD8 T Subtypes

**Figure 4:**
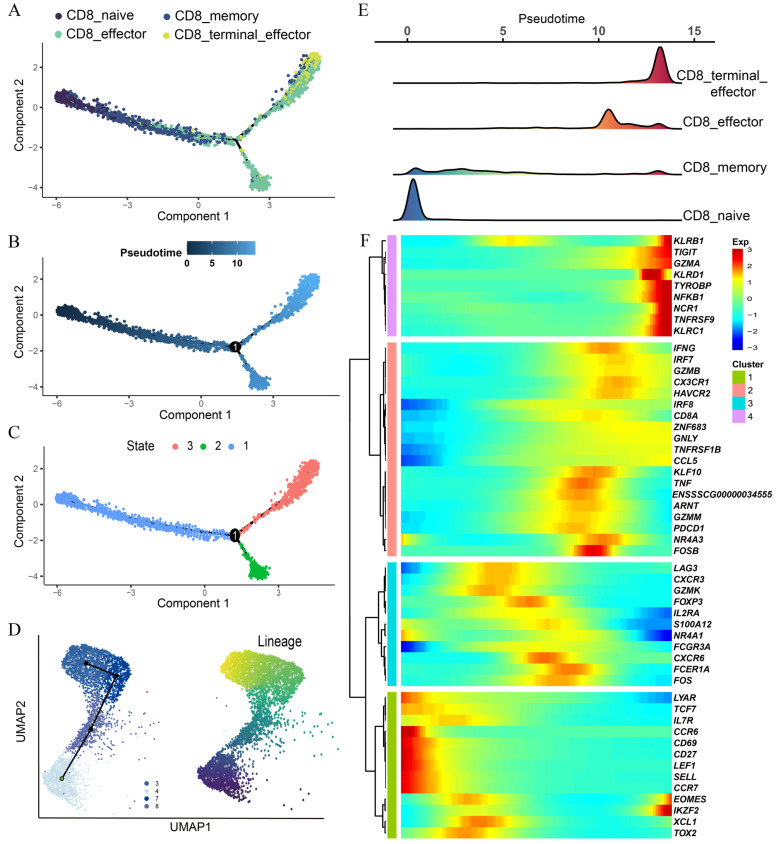
Developmental trajectory analysis of porcine CD8 T cells. (**A**) Transformation among CD8 T cell subtypes predicted by Monocle2. The rainbow colors from blue to yellow represent the trajectory from the beginning to the end. (**B**,**C**) Pseudotime trajectories of CD8 T cells inferred by Monocle2. Cells are colored according to pseudotime (**B**) and cell states (**C**). (**D**) CD8 T cell differentiation trajectory inferred by Slingshot according to cluster information (left) and pseudotime (right). (**E**) Ridge plot of CD8 T cell subtypes over pseudotime. (**F**) Heatmap of relative expression of representative marker genes in CD8 T cell subtypes along the inferred trajectory.

Further, we conducted single-cell trajectory analysis to investigate the developmental status of CD8 T cell subtypes with the highest heterogeneity using the Monocle2 (v2.22.0) and the SlingShot software packages (v2.2.1). The results revealed that a majority of cells from each cluster gathered based on the gene expression similarity and that the CD8 T cell subtypes formed a relatively continuous pseudo-time trajectory which began with the CD8_naive cells, followed by CD8_memory, and then branched into two different trajectories represented by CD8_effector and CD8_terminal_effector, respectively (Figure 4A–C). The pseudo-time heatmap showed that CD8_memory appeared to be an intermediate state between naive and effector T cells (Figure 4A,E). Moreover, CD8_effector and CD8_terminal_effector T cells exhibited higher pseudo-time scores than two other CD8 T cell subtypes, indicating their terminal developmental state (Figure 4B). Furthermore, we also inferred the progression of the cellular transcriptomes using Slingshot. As expected, Slingshot analysis confirmed the inferred pseudotime trajectory, which started with CD8_naive serving as the root node and transitioned from the CD8_memory to CD8_effector and CD8_terminal_effector cells (Figure 4D). According to the transition processes, genes were clustered into four modules, which were highly consistent with the developmental trajectories of CD8 T cell subtypes we defined (Figure 4F). For example, genes such as *LEF1*, *SELL*, and *TCF7* were specifically expressed in CD8_naive, and CD8_memory showed high expression of genes, such as *GZMK*, *CXCR3*, and *FCGR3A*. Genes such as *GZMB*, *GNLY*, and *GZMM* were highly expressed in CD8_effector, and most NK cell markers, including *NCR1*, *KLRC1*, *NFKB1*, and *KLRB1* were specifically expressed in CD8_terminal_effector. Overall, our study has clearly defined major developmental trajectories of CD8 T cell subtypes, which provide valuable insights into the biological characteristics of CD8 T cells in the context of porcine immune responses.

### 3.5. Single-Cell Network Inference and Cell Communication Analysis Unveil Candidate Regulators and Extensive Intercellular Communication in CD8 T Subtypes

**Figure 5:**
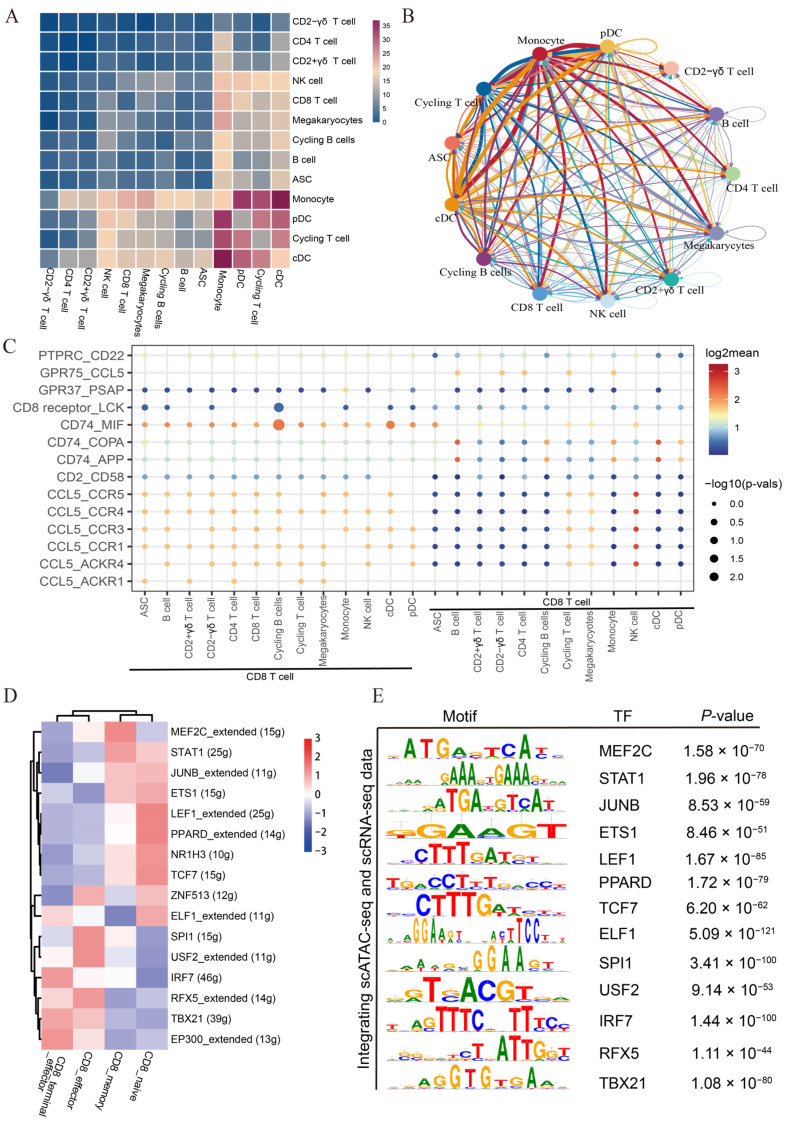
Cell communication between CD8 T cells and other cell types and SCENIC analysis of CD8 T cell subtypes. (**A**) Heatmap of the number of significant ligand-receptor interactions among different cell types. (**B**) The interaction network of immune cell types. (**C**) Dot plot of significant ligand-receptor pairs involved in the interaction between CD8 T cells and other cell types. Dot size represents the significance level (−log10(*p*-value)), while colors indicate the expression levels (log2 mean (molecule 1−molecule 2)). (**D**) Heatmap of transcription factor regulon activity in CD8 T cell subtypes. (**E**) Motif enrichment analysis in CD8 T cells by integrating scATAC-seq and scRNA-seq data.

We applied the CellphoneDB algorithm to predict cell-cell receptor-ligand interactions between CD8 T cells and other cell types (CD4 T cells, B cells, monocytes, dendritic cells, and NK cells) in the blood microenvironment. The results showed that monocytes exhibited the most interactions with other cell types, and the strongest interactions were observed between monocytes and dendritic cell types (cDC and pDC) (Figure 5A,B). The interaction signal strength is shown in Figure 5A. In the CD74 signaling network, a significant interaction occurred between B cells, monocytes, or dendritic cells and CD8^+^ T cells through related receptor-ligand pairs, including CD74-APP and CD74-COPA (Figure 5C). In CD8 T cells, the chemokine CCL5 was highly expressed, whose receptors include CCR5, CCR4, CCR3, CCR1, ACKR1, and ACKR4. The majority of these receptors were important in immune regulation, and CD8 T cells primarily interacted with several other cell types through the specific ligand-receptor pairs composed of CCL5 and these receptors. (Figure 5C). In addition, CD8 T cells also interacted with all other cell types through another common CD74-MIF pair in pig peripheral blood. The CD74-MIF pair has been reported to modulate immune activity, and CD74 is an important receptor regulating dendritic cell migration and immune response, and it also is involved in regulating T cell and B cell development [31,32]. In addition, we also explored the communication network among CD8 T cell subtypes. We found that CD8_terminal_effector cells frequently interacted with the other three CD8 T cell subtypes through the receptor-ligand pairs, further suggesting the important crosstalk effects of CD8_terminal_effector (Figure S4A,B).

Transcription factors (TFs) play an important role in regulating gene expression and shaping different phenotypes of T cells [33]. Thus, we employed SCENIC to identify potential differential TFs from CD8 T cell subtypes. We identified some highly active TFs in each CD8 T cell subtype. For example, TFs *LEF1*, *TCF7*, *PPARD*, and *NR1H3* were highly active in CD8_naive cells, TFs *STAT1* and *MEF2C* in CD8_memory cells, *SPI1*, *USF2*, and *RFX5* in CD8_effector cells, and *IRF7*, *TBX21*, and *EP300* in CD8_terminal_effector cells (Figure 5D). Meanwhile, in conjunction with the published PBMC scATAC-seq data [25], further analysis revealed that the TFs predicted in CD8 T cells through scRNA-seq were also significantly enriched in motif enrichment analysis for the ATAC peaks specific to the CD8^+^ T cluster (Figure 5E). These findings collectively suggest that multiple TFs synergistically regulate T cell development and maintain the heterogeneity of CD8 T cells. Taken together, the above results elucidate the possible molecular basis of cell-cell interactions in the peripheral blood and reveal the underlying mechanism of the CD8 T cell phenotypic switch.

### 3.6. Cross-Species Comparison Shows Similarities and Differences between Species

We integrated previously published PBMC datasets from six healthy adult humans and seven healthy dogs with the PBMC datasets from eight healthy pigs in our study [15,26]. Our results indicated that there were more similarities than differences between species. For instance, porcine dendritic cells (pDCs and cDCs), B cells, monocytes, ASC cells, and erythrocytes clustered together with their corresponding cell types in humans and dogs on the same evolutionary branches (Figure S5A,B). Porcine CD4_naive defined in this study corresponds to naive_CD4 cells in both humans and dogs, and our defined porcine CD4_TEM corresponds to Tm_CD4 cells in humans and Tem_CD4 cells in dogs. Notably, we found subtle differences between species. For example, two subtypes of porcine γδ cells clustered separately with two subtypes of canine γδ cells, but porcine γδ cells and human γδ cells were on different branches. These results suggested that pigs and dogs belonged to the high γδ cell species and that their γδ cells were evolutionarily closer to each other but more distant from human γδ cells. Our defined CD8_effector and CD8_terminal_effector cell types were closer to human NK cells, which was similar to one previous report that canine CD8 effector T cells clustered together with human NK cells [15]. Furthermore, porcine CD8_memory cells and human CD8_memory cells clustered on the same evolutionary branch. CD8_naive cells were not available in human data, but we observed that porcine CD8_naive cells and canine CD8_naive cells shared a common branch. In summary, although cross-species analysis underscored similarities among immune cells, it also highlighted potential inter-species differences.

## 4. Discussion

CD8 T cells play a pivotal role in adaptive immune responses against intracellular pathogens. Currently, CD8 T cells have been extensively studied in human and mouse models, but knowledge of porcine cytotoxic T lymphocytes (CTLs) remains limited. In this study, we first characterized the heterogeneity of peripheral blood circulating immune cells through the transcriptome analysis of 34,220 single cells based on multiple datasets from our experiment and open databases. We newly identified four CD8 T cell subtypes, and they exhibited different transcriptomic profiles and pathway activities in peripheral blood. Additionally, we revealed potential differentiation trajectories of four CD8 cell subtypes by Monocle2 and Slingshot analyses. Finally, we inferred the interactions between CD8 T cells and other immune cells and identified key transcription factors (TFs) involved in the formation and maintenance of different T cell phenotypes.

In our study, we identified two distinct subtypes of γδ T cells based on the expression levels of the *TRDC* and *CD2* genes. Comparison between human and pig data revealed that γδ T cells from these two species fell into separate branches. In contrast, the comparison of pig and dog data found a high similarity in their γδ T cells. Specifically, dog γδ T cells and pig CD2^−^γδ T cells clustered in the same clade, and pig CD2^+^γδ T cells (highly expressing *CD8A*) and dog CD8^+^γδ T cells clustered in the same clade. Further, GO enrichment analysis revealed functional differences between CD2^+^γδ T cells and CD2^−^γδ T cells in pigs. CD2^−^γδ T cells primarily participated in immune system regulation, while CD2^+^γδ T cells were mainly involved in cell migration processes. Another interesting finding was that naive CD8 T cells were closer to naive CD4 T cells than other CD8 T cells in the UMAP plot. This observation was consistent with previous findings of dog PBMCs [15]. This unique clustering pattern of T cells might be attributed to the fact that naive CD8 T cells have not encountered specific antigens yet, and thus, they exhibit no cytotoxicity. Furthermore, our clustering tree showed that the two distinct effector CD8 T cell subtypes were phylogenetically closer to human NK cells, indicating potential interspecies differences. Our data indicated that in the case of investigating non-model species, various methods should be combined, and multiple databases should be integrated for cell type annotation.

Although the various transitions of T cells are generally considered to occur primarily in lymph nodes and/or infection sites, a recent scRNA-seq study of healthy humans has demonstrated the existence of a clear cell developmental trajectory in peripheral blood, where naive T cells (T_N_ cells) differentiate into memory T cells (T_CM_ and T_EM_ cells) and further differentiate into CD8^+^ tissue-resident memory (T_RM_) cells [34]. Similar differentiation trajectories were identified when studying the dynamic relationships among T cell subtypes across tissues in adults, which revealed that certain CD8^+^ T cells underwent clonal expansion in multiple tissues at different developmental stages and that diverse T cells were widely distributed throughout the human body through clonal expansion and transformation [35]. In order to explore the transformation relationships among porcine CD8 T cell subtypes in peripheral blood, we conducted a pseudo-time trajectory analysis. We found that CD8_naive cells and CD8_memory primarily clustered along the pseudotime axis, while CD8_effector and CD8_terminal_effector diverged in two directions. Interestingly, effector CD8 T cells appeared in two directions and were more dominant in state 2 (CD8_effector), whereas CD8_terminal_effector T cells were mainly present at the end of state 3. This phenomenon is similar to the asymmetric cell fate model of human effector CD8 T cells [36]. This model suggests that human effector CD8 T cells in intermediate differentiation states undergo asymmetric cell division upon repeated encounters with antigen-presenting cells (APCs) [36]. This process allows some activated T cells to collectively receive strong differentiation signals, driving them toward terminal differentiation. Simultaneously, this process preserves the great potential of less differentiated cells to develop into memory cells [36]. In sum, our trajectory analysis revealed that CD8_naive was the starting point for differentiation and CD8_memory was an intermediate transitional state, or CD8_effector T cells dedifferentiated into memory cells with features of naive and effector T cells [37,38].

In addition to trajectory analysis, we also conducted GO analysis and GSVA analysis to investigate the potential biological functions and pathway activities of different CD8 T cell subtypes. Our GO analysis revealed that the biological functions of CD8_naive cells were largely associated with ribosome synthesis, including “ribosome biogenesis”, “rRNA processing”, and “ribosomal large subunit assembly”. Our finding was in line with one previous report that a substantial pool of ribosomal subunits is present in naive T cells and that these ribosomes can rapidly participate in the T cell activation process upon antigen stimulation [39]. Our GO analysis also showed that memory CD8 T cells and effector CD8 T cells were mainly related to the immune response biological processes, such as “regulation of immune system process”, “adaptive immune response”, and “leukocyte activation”. Furthermore, we also found that CD8_effector cells were extensively involved in the regulation of actin filament-related biological processes, and actin filaments have been demonstrated to play a crucial role in T cell immune synapse formation and T cell activation [40]. CD8_terminal_effector T cells at the terminal differentiation stage were extensively involved in oxidative phosphorylation and ATP metabolism processes. In addition, our GSVA (hallmark gene sets) and KEGG analyses also revealed the complex pathway heterogeneity of CD8 T cells in the peripheral blood.

Cells often communicate with each other to coordinate their behavior [41]. In this study, we investigated the interactions between CD8 cells and other cells in peripheral blood by CellPhoneDB analysis. We found that most CD8 T cells communicated with other cells through the CCL5-related pairs (CCL5 ligand and CCR5, CCR4, CCR3, CCR1, ACKR1, and ACKR4 receptors). Notably, CCL5 has been reported to be a key effector molecule of T lymphocytes, playing a role in regulating the migration of T lymphocytes and monocytes [42,43]. We also found that several types of cells with antigen-presenting ability, such as monocytes, dendritic cells, and B cells, utilized CD74-APP and CD74-COPA pairs to interact with CD8 T cells, thereby promoting T cell activation and polarization. Transcription factors have been reported to participate in the formation and maintenance of different T cell phenotypes [33]. Further, we identified TFs associated with cell differentiation using SCENIC. Consistent with the differential gene expression analysis results, the greatest differences in transcription factor activity were observed between CD8_terminal_effector cells and CD8_naive T cells that were highly expressing *LEF1*, *TCF7*, *PPARD*, and *NR1H3*. Previous research has established the essential roles of WNT signaling pathway effectors, *LEF1* and *TCF7*, in the activation and quiescence of peripheral CD8_naive T cells [44,45,46]. CD8_memory cells prominently expressed *MEF2C* and *STAT1*. Interestingly, CD8_naive and CD8_memory cells shared *JUNB* and *ETS1*, suggesting a potential lineage relationship between them, with CD8_memory possibly originating from naive CD8 T cells. *SPI1*, encoding PU.1, has been reported to control T cell differentiation [47]. CD8_effector cells lacked *TCF7* regulon activity but displayed high *SPI1*, *USF2*, and *RFX5* regulon activities. Transcription factors *IRF7* and *TBX21* (T-bet) have been found to be associated with CD8 T cell cytotoxicity [48]. In this study, we found that these two TFs had high activities in CD8_terminal_effector T cells. Furthermore, we also observed several less-reported transcription factors, such as *PPARD* and *ELF1*, in various subtypes of CD8 T cells, and these TFs might potentially play a role in the differentiation and phenotypic maintenance of different T cells. Recently, a scATAC-seq study utilizing PBMCs from Duroc pigs predicted TFs for various immune cell subtypes, including CD8 T cells [25]. Through our analysis, we found that TFs identified in our study were also significantly enriched in the scATAC-seq study. This result strongly supported the reliability of the identified TFs in CD8 T cells. In summary, we identified known and unknown TFs that might contribute to phenotypic heterogeneity of CD8^+^ T cells. However, there were some limitations of this study, such as the lack of experimental validation of the cell subsets or marker genes we identified.

## 5. Conclusions

In conclusion, our analysis of porcine peripheral blood scRNA-seq data has defined four different CD8 T cell subtypes and revealed their functional and dynamic heterogeneity. Through single-cell regulatory network inference, we identified key regulatory factors during CD8 T cell differentiation. Moreover, cell-cell communication analysis underscored extensive intercellular interactions among different cell types. Lastly, our cross-species analysis highlighted both similarities and potential differences between species. Our findings provide insights into the diverse CD8 T cell state transitions in peripheral blood and identify core regulators of CD8 T cell identity, enriching our understanding of the porcine immune system.

## Data Availability

Raw sequencing data (FASTQ format) and processed data files are available in the NCBI Gene Expression Omnibus database under accession number GSE247126.

## Supplementary Materials

The following supporting information can be downloaded at: https://www.mdpi.com/article/10.3390/cells13080692/s1, Figure S1. 32 cell clusters in porcine peripheral blood mononuclear cells obtained by unsupervised clustering. (A) The UMAP plot of integrated transcriptome of 34,220 single cells derived from all 8 PBMC samples. Color brightness represents sample source of cells. (B) Stacked bars of 32 cell clusters. The bar size represents the percentage of 8 PBMC samples in each cluster. (C) Hierarchical clustering trees of the relationships among cell clusters; Figure S2. SingleR comparison between procine PBMCs and human PBMCs and the percentage of PBMC samples in each of 15 cell types. (A–E) Cell types were annotated using SingleR based on their similarity to human PBMC. The 5 human reference datasets utilized in this study are HumanPrimaryCellAtlasData (ref1), BlueprintEncodeData (ref2), DatabaseImmuneCellExpressionData (ref3), NovershternHematopoieticData (ref4), and MonacoImmuneData (ref5). (F) Stacked bars of 15 major cell types. The bar size indicates the percentage of PBMC samples in each of 15 major cell types; Figure S3. Transcriptome heterogeneity in T lymphocytes. (A) Sankey plot of initial clusters and subset clusters. Clusters with the prefix “S” indicate the subset cluster. “S9” corresponds to the “low-quality cluster”. (B) Stacked bar. Bar size indicates the proportions of PBMC samples in each of 10 major T cell types. (C) Volcano plot of the DEGs (pseudobulk conversion) between CD4_naive and CD4_TEM (Supplemental Table S2). Adjusted *p* values < 0.05 and |log_2_ FC| > 0.58 were used as DEG screening criteria. Red and blue dots represent up-regulated and down-regulated DEGs, respectively. (D) GO analysis of down-regulated and up-regulated DEGs obtained by pseudobulk conversion (Supplemental Table S3). Blue and orange bars indicate the pathways enriched with up-regulated and down-regulated DEGs, respectively. (E) Volcano plot of DEGs (pseudobulk conversion) between CD2^−^γδ and CD2^+^γδ T cells (Supplemental Table S2). Red and blue dots represent up-regulated and down-regulated DEGs in the CD2^−^γδ cluster, respectively. (F) GO analysis of up-regulated and down-regulated DEGs obtained by pseudobulk conversion (Supplemental Table S3). Blue and orange bars denote the pathways enriched with up-regulated and down-regulated DEGs, respectively; Figure S4. Cell communication among CD8 T cells subtypes. (A) Heatmap of the number of significant ligand-receptor interactions among different CD8 T cell subtypes. CD8_C1 represents CD8_naive cell; CD8_C2 represents CD8_memory cell; CD8_C3 represents CD8_effector cell; and CD8_C4 represents CD8_terminal_effector cell. (B) Dot plot of the significant ligand-receptor pairs involved in the interaction among CD8 T cell subtypes. Dot size represents the significance level (−log10(*p*-value)), while colors indicate the expression levels (log2 mean (molecule 1−molecule 2)); Figure S5. Comparison between porcine PBMCs and human PBMCs or dog PBMCs. (A) Hierarchical clustering of human and pig cell types using the top 2000 variable features after SCT normalization. (B) Hierarchical clustering of pig and dog cell types using the top 2000 variable features after SCT normalization. The prefix “hu_” represents human cell types; Supplemental Table S1. Differentially expressed genes among CD8 T subtypes; Supplemental Table S2. Differentially expressed genes between CD4^+^ T cell subtypes and γδ cell T subtypes; Supplemental Table S3. Gene Ontology enrichment analysis for CD4^+^ T cell subtypes and γδ T cell subtypes; Supplemental Table S4. The results of GO analysis, pathway activity analysis, and KEGG analysis among CD8 T subtypes.
