# Supplementary material for: Single-Cell RNA-Sequencing Reveals Heterogeneity and Transcriptional Dynamics in Porcine Circulating CD8+ T Cells
Source: Cells. 2024 Apr 16;13(8):692. doi: 10.3390/cells13080692 (PMC11049515; doi:10.3390/cells13080692)
Supplement: Supplementary file 1 [file cells-13-00692-s001.zip › Supplementary Figures.pdf]

Supplementary Materials

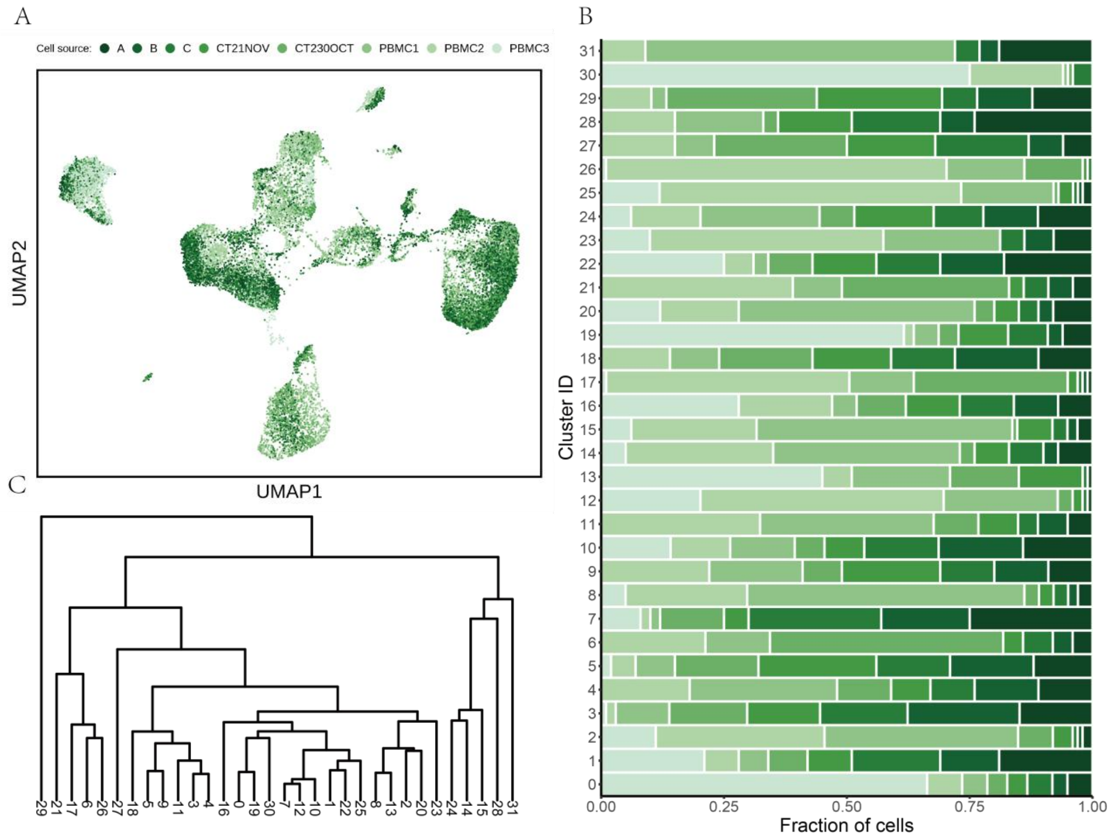

Figure S1. 32 cell clusters in porcine peripheral blood mononuclear cells obtained by unsupervised clustering. (**A**) The UMAP plot of integrated transcriptome of 34,220 single cells derived from all 8 PBMC samples. Color brightness represents sample source of cells. (**B**) Stacked bars of 32 cell clusters. The bar size represents the percentage of 8 PBMC samples in each cluster. (**C**) Hierarchical clustering trees of the relationships among cell clusters.

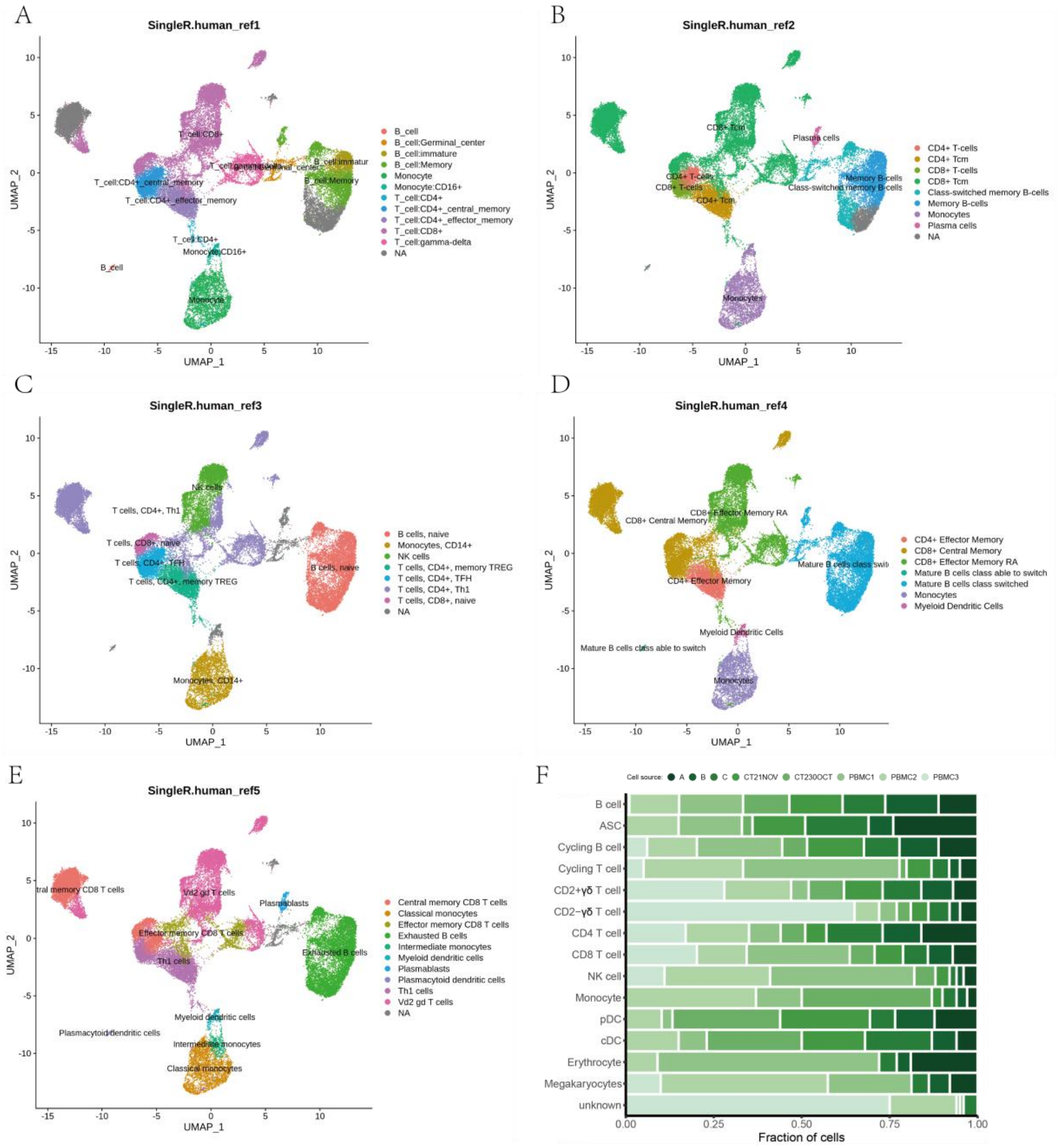

Figure S2. SingleR comparison between procine PBMCs and human PBMCs and the percentage of PBMC samples in each of 15 cell types. (**A-E**) Cell types were annotated using SingleR based on their similarity to human PBMC. The 5 human reference datasets utilized in this study are HumanPrimaryCellAtlasData (ref1), BlueprintEncodeData (ref2), DatabaseImmuneCellExpressionData (ref3), NovershternHematopoieticData (ref4), and MonacoImmuneData (ref5). (**F**) Stacked bars of 15 major cell types. The bar size indicates the percentage of PBMC samples in each of 15 major cell types.

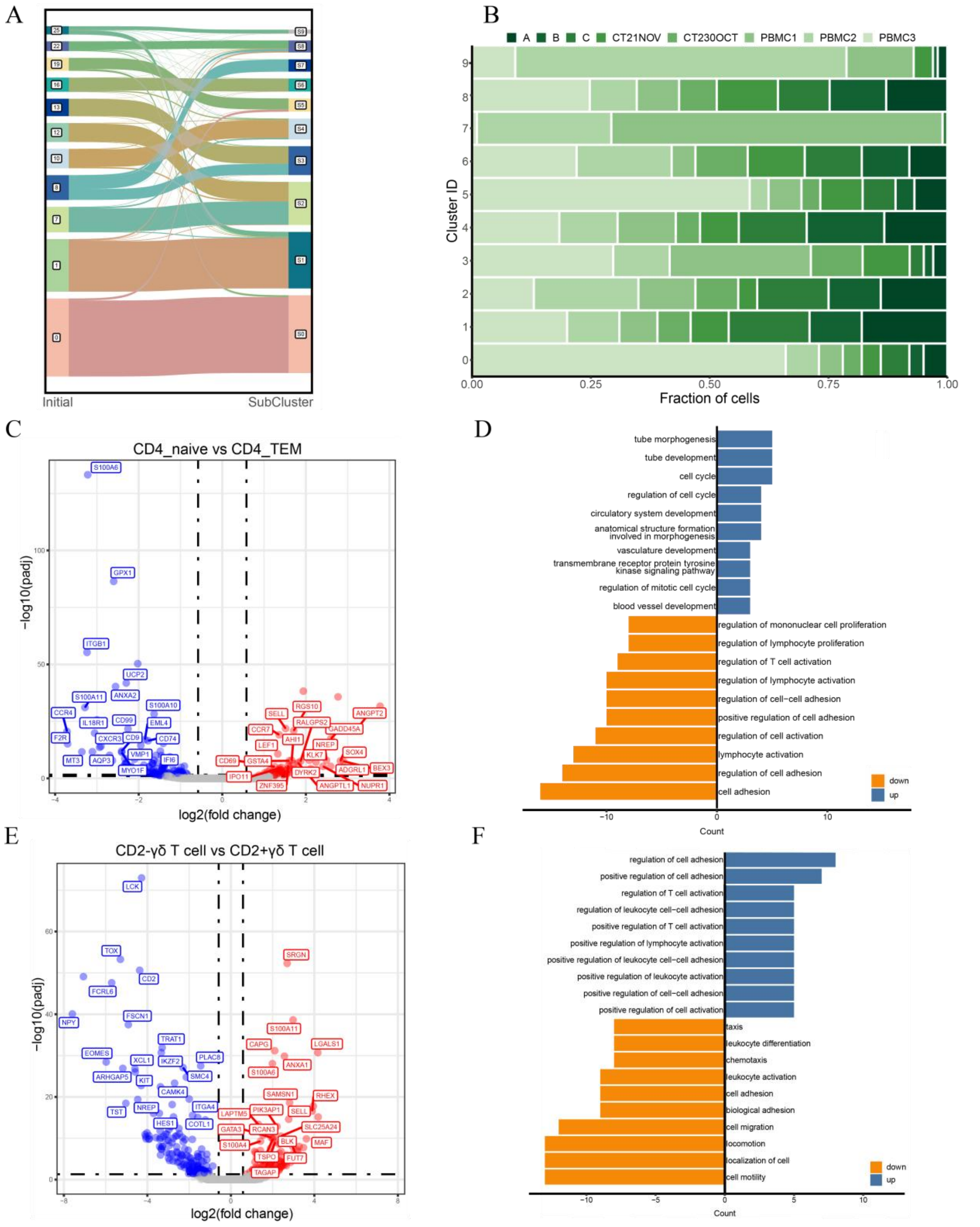

Figure S3. Transcriptome heterogeneity in T lymphocytes. (**A**) Sankey plot of initial clusters and subset clusters. Clusters with the prefix “S” indicate the subset cluster. “S9” corresponds to the “low-quality cluster”. (**B**) Stacked bar. Bar size indicates the proportions of PBMC samples in each of 10 major T cell types. (**C**) Volcano plot of the DEGs (pseudobulk conversion) between CD4_naive and CD4_TEM (Supplemental Table Ⅱ). Adjusted P values < 0.05 and $|\log_2 FC| > 0.58$ were used as DEG screening criteria. Red and blue dots represent up-regulated and down-regulated DEGs, respectively. (**D**) GO analysis of down-regulated and up-regulated DEGs obtained by pseudobulk conversion (Supplemental Table Ⅲ). Blue and orange bars indicate the pathways enriched with up-regulated and down-regulated DEGs, respectively. (**E**) Volcano plot of DEGs (pseudobulk conversion) between $CD2^{-}\gamma\delta$ and $CD2^{+}\gamma\delta$ T cells (Supplemental Table Ⅱ). Red and blue dots represent up-regulated and down-regulated DEGs in

the CD2⁻γδ cluster, respectively. (**F**) GO analysis of up-regulated and down-regulated DEGs obtained by pseudobulk conversion (Supplemental Table Ⅲ). Blue and orange bars denote the pathways enriched with up-regulated and down-regulated DEGs, respectively.

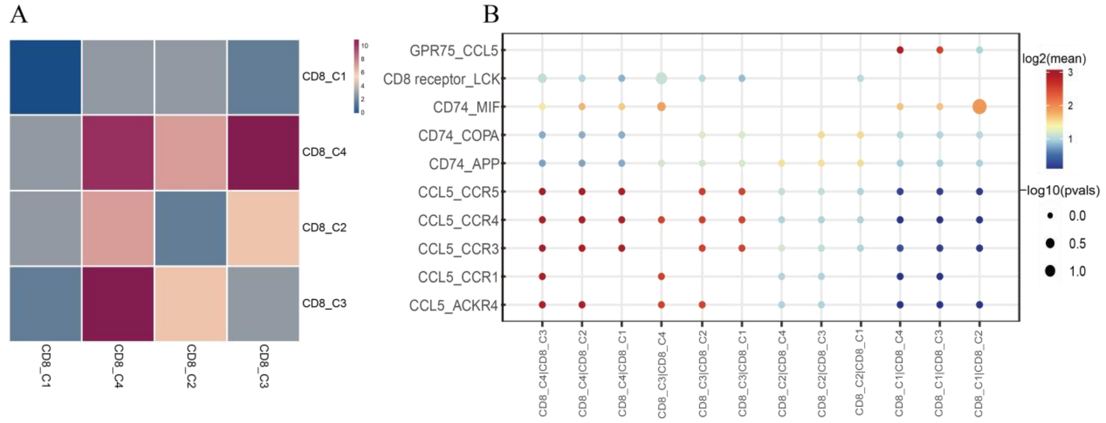

Figure S4. Cell communication among CD8 T cells subtypes. (**A**) Heatmap of the number of significant ligand-receptor interactions among different CD8 T cell subtypes. CD8_C1 represents CD8_naive cell; CD8_C2 represents CD8_memory cell; CD8_C3 represents CD8_effector cell; and CD8_C4 represents CD8_terminal_effector cell. (**B**) Dot plot of the significant ligand-receptor pairs involved in the interaction among CD8 T cell subtypes. Dot size represents the significance level (−log10(p-value)), while colors indicate the expression levels (log2 mean (molecule 1−molecule 2)).

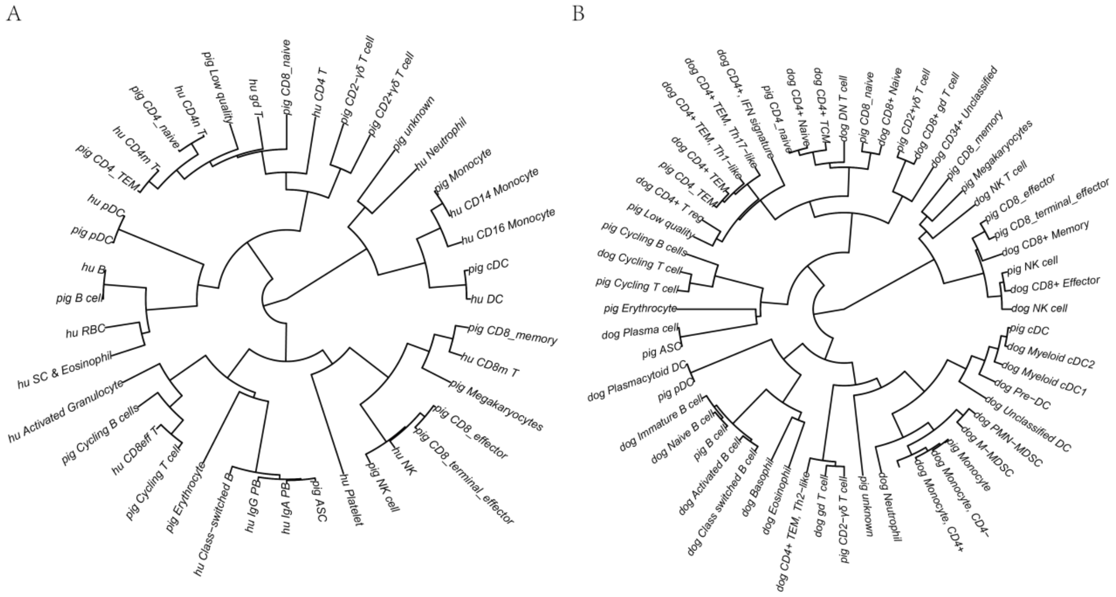

Figure S5. Comparison between porcine PBMCs and human PBMCs or dog PBMCs. (**A**) Hierarchical clustering of human and pig cell types using the top 2000 variable features after SCT normalization. (**B**) Hierarchical clustering of pig and dog cell types using the top 2000 variable features after SCT normalization. The prefix “hu_” represents human cell types.
